# The resurgence of yellow fever outbreaks in Nigeria: a 2-year review 2017–2019

**DOI:** 10.1186/s12879-021-06727-y

**Published:** 2021-10-11

**Authors:** Terna Nomhwange, Anne Eudes Jean Baptiste, Obi Ezebilo, Joseph Oteri, Lois Olajide, Kizito Emelife, Shehu Hassan, Erdoo R. Nomhwange, Kennedy Adejoh, Faith Ireye, Eyo E. Nora, Adamu Ningi, Blaise Bathondeli, Oyewale Tomori

**Affiliations:** 1 World Health Organization, Country Office, Abuja, Nigeria; 2 United Nations Children’s Fund, Abuja, Nigeria; 3 National Primary Health Care Development Agency, Abuja, Nigeria; 4 Nigeria Centre for Disease Control, Abuja, Nigeria; 5 University of Abuja Teaching Hospital, Abuja, Nigeria; 6 World Health Organization-Nigeria, Abuja, Nigeria; 7 World Health Organization Regional Office for Africa, Brazzaville, Congo; 8 Independent Consultant and Professor of Virology, Abuja, Nigeria

**Keywords:** Yellow fever, EYE strategy, Arboviruses, VPD outbreaks, Vaccination

## Abstract

**Background:**

Yellow fever outbreaks are documented to have a considerable impact not only on the individuals but on the health system with significant economic implications. Efforts to eliminate yellow fever outbreaks globally through the EYE strategy remains important following outbreaks in Africa, Nigeria included. The outbreaks reported in Nigeria, since 2017 and the response efforts provide an opportunity to document and guide interventions for improving future outbreaks in Nigeria and other countries in Africa.

**Methods:**

We reviewed the available yellow fever surveillance and vaccination response data between September 2017 and September 2019 across the 36 states across Nigeria. We described the epidemiology of the difference outbreaks and the periods for all interventions. We also documented the emergency vaccination responses as well as preventive mass vaccinations implemented towards improving population immunity and limiting epidemic potentials in Nigeria.

**Results:**

A total of 7894 suspected cases with 287 laboratory-confirmed cases were reported in Nigeria between September 2017 and September 2019 with a mean age of 19 years and a case fatality of 2.7% amongst all reported cases. Outbreaks were confirmed in 55 LGAs with most of the outbreaks across four major epicentres in Kwara/Kogi, Edo, Ebonyi and Bauchi states. In response to these outbreaks, eight reactive vaccination campaigns, supported through ICG applications, were implemented. The duration for responding to the outbreaks ranged from 15 to 132 days (average 68 days) and a total of 45,648,243 persons aged < 45 years vaccinated through reactive and preventive mass campaigns between September 2017 and September 2019**.**

**Conclusions:**

Nigeria experienced intermediate outbreaks of yellow fever between September 2017 and 2019 with vaccination responses conducted to control these outbreaks. However, there are delays in the timeliness of responses and more efforts required in improving reporting, response times and preparedness to further prevent morbidity and mortality from the yellow fever disease outbreaks. These efforts, including improving routine yellow fever coverage, contribute towards improving population immunity and other activities related to achieving the goals of the EYE strategy.

## Introduction

Yellow fever is a viral haemorrhagic fever transmitted by the *Aedes* Mosquito sp. and has been reported in African and the Americas since the twentieth century. With outbreaks documented to have a considerable impact not only on the individuals but on the health system with significant economic implications [1]. This disease is vaccine-preventable with the 17D yellow fever vaccine available and used globally since the 1930s [2–4]. This vaccine is known to confer protection in 95% of cases within 30 days and to provide life long immunity in all persons who have been vaccinated [5, 6].

Yellow fever outbreaks are sustained in forested areas through sylvatic (i.e., between non-human primates and mosquitoes) and intermediate transmission (i.e., from mosquitoes to humans) [7]. Urban amplification tends to occur in densely populated urban and peri-urban centres between humans and urban mosquitoes. Outbreaks are reported following cases of suspected yellow fever fitting standard case definition and confirmation by laboratory techniques through serology, plaque reduction and neutralisation test (PRNT) or real-time polymerase chain reaction (PCR-rt). However, challenges with serology include cross-reactivity with other flaviviruses [8].

The Integrated Disease Surveillance and Response (IDSR) strategy is used in the African region to guide support to countries through improving surveillance and preparedness towards improving the quality of response to outbreaks of epidemic-prone diseases [9]. Transmission risk continues to increase with suboptimal immunisation coverage and increasing urbanisation noted within the African region, Nigeria inclusive [10]. The yellow fever vaccine was introduced into the routine immunization schedule of Nigeria in 2004 with coverage estimates of 65% from 2016 to 2018 through the national immunisation coverage surveys (NICs) and World Health Organisation (WHO) and United Nations Children’s Fund (UNICEF) estimate for immunisation coverage (WUENIC) [11]. The challenges in yellow fever vaccine coverage are highlighted by the negative difference in coverage between measles and yellow fever vaccine offered at the same time, most countries [12]. Shearer et al., have documented these gaps in Nigeria and other countries through a review of yellow fever vaccination coverage between 1970 and 2016 [13]. As of 2018, Nigeria alone accounted for almost a third of persons at risk of yellow fever, with a conservative estimate of 112 million individuals in all districts still requiring yellow fever vaccination [14].

Nigeria is regarded as one of the high-risk countries for yellow fever transmission and a high priority for implementation of the eliminating yellow fever epidemics (EYE) strategy. Outbreaks have been documented in Nigeria, with the largest outbreaks reported between 1985 and 1991 with over 40,000 suspected cases reported [15, 16]. In 2016, outbreaks were reported in Angola and Uganda with international spread to China [17]. Following challenges with the global supply of yellow fever vaccines and the need to address yellow fever as a public health concern, the Eliminating Yellow Fever Epidemics (EYE) strategy was launched in Nigeria in April 2018 with three strategic objectives [18].

The increasing number of outbreaks and persons affected in Nigeria remains a concern for the country as well as the African region with increasing effects on the health system and substantial financial implications for yellow fever control.

We described the epidemiology of yellow fever disease in Nigeria following the outbreaks between September 2017 to September 2019 and assessment of the response to these outbreaks.

## Methods

We conducted a retrospective descriptive study of the epidemiology of the yellow fever outbreak in Nigeria over a 2-year period between September 2017 and September 2019.

### Study area

Nigeria is located in the West African on the Gulf of Guinea between Benin and Cameroun with an estimated population in 2019 of 198 million persons as projected from the 2006 Census. Nigeria covers an area of 923,768 km^2^ with savannah and forest vegetation in the northern and southern parts with climatic conditions which promote spread of arthropods. Forest reserves make up about 10% of the total land area in Nigeria, with a reported population of primates [19].

In Nigeria, there are four designated laboratories for yellow fever testing within the WHO network and the National Reference Laboratory in Abuja. Samples are preliminarily tested via serology and IgM positive cases considered as probable cases. Samples for all probable cases are subsequently shipped to the Regional Reference Laboratory located at the Institute Pasteur, Dakar Senegal where confirmation is done via repeat serology and subsequent real-time polymerase chain reaction (PCR-rt) and plaque reduction neutralisation tests (PRNT). Results from RRL Institute Pasteur, Dakar are shared with Nigeria routinely after the tests are conducted.

All confirmatory results received in the country at national levels are officially communicated immediately to the states. The state teams subsequently conduct, an outbreak investigation with documentation of clinical features, vaccination status, travel history and other variables. Entomological surveys are also documented to assess the presence of the vector and the risk of amplification and urban spread.

An outbreak report is subsequently developed and based on the recommendations, an International Coordination Group (ICG) request completed to support reactive vaccination campaigns to control the spread. All vaccination campaign data are collated, and post-campaign surveys conducted to validate the quality of the campaign.

In September 2017, a yellow fever case was confirmed in a 12-year-old girl in Ifelodun Local Government Area (LGA) of Kwara state [20]. This outbreak spread across multiple states from September 2017 to October 2019. Eleven reactive vaccination campaigns covering 65 LGAs were conducted from September 2017 to October 2019. Also, the national laboratory network was expanded from four to seven laboratories for preliminary testing by serology. Other interventions included the activation of the incident management system (IMS) and functional public health emergency operation centres (PHEOCs) were also in place to support the outbreak response.

### Subjects

We reviewed the country programme data with a specific focus on the immunisation and surveillance and immunisation country database reported by all 36 states plus the Federal Capital Territory (FCT) Abuja and the 774 LGAs maintained by the Government of Nigeria and supported by the World Health Organisation at national levels.

### Measurements

We measured and compared the number of suspected and confirmed cases of yellow fever reported by LGA and state levels throughout the review as well as the number of Interventions conducted in response to these outbreaks. We also tracked the trends of these outbreaks over time to describe the epidemiological patterns. Yellow fever cases were also summarised by age and sex distribution as well as case fatality rates patterns across states between September 2017–2019.

**Fig. 1:**
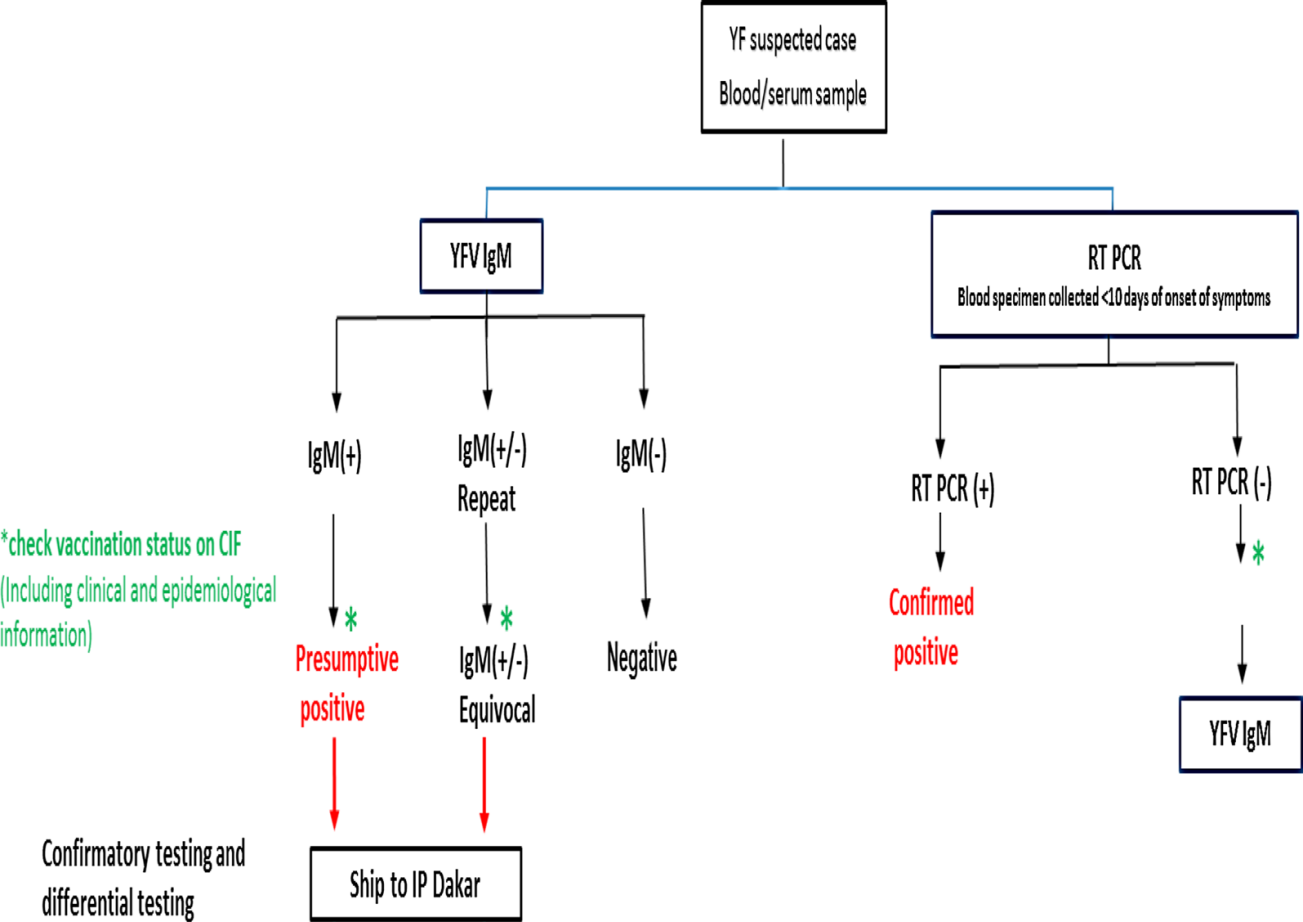
Algorithm for the confirmation of yellow fever cases in Nigeria

Yellow fever surveillance in Nigeria is implemented as a case-based surveillance with information on all suspected cased reported and collated at LGA, State and National levels. Based on the yellow fever surveillance national guidelines, suspected cases which fit the standard case definition are investigated with blood samples collected for laboratory confirmation as displayed in Fig. 1.

We reviewed the data of the eleven reactive vaccination campaigns covering 65 LGAs which were conducted from September 2017 to October 2019 and other interventions put in place to support the outbreak response. These included national laboratory network expansion from four to seven laboratories for preliminary testing by serology and activation of the incident management system (IMS) and functional public health emergency operation centres (PHEOCs).

### Data management

All programme data were collated through a Microsoft Access® database collected at National and sub national levels and updated weekly. Immunisation data was also collected through the same system and cleaned following regular data harmonisation conducted by the Data Management Team within WHO and the Nigeria Centre for Disease Control (NCDC). Immunisation data are managed by the National Primary Health Care Development Agency (NPHCDA) and supported by WHO. Entomological reports, detailed case investigations forms and Laboratory data were also reviewed and summarised for variables of interests.

Non-available data variables or lost data was accounted for, and assumptions for entries/replacements stated accordingly.

All available data were analysed using Microsoft Access and Excel and presented as frequencies, proportions and trends.

### Ethics

No ethical standards were bridged during the process of this review. All data used were accessed from the available yellow fever surveillance and immunisation programme from the National primary healthcare development agency (NPHCDA) and Nigeria centre for disease control (NCDC) through the Yellow fever technical working group (TWG).

## Results

In total, 4528 (57%) of the 7894 suspected cases reported from September 2017 to October 2019 were male; reporting a male to female ratio of 1.3:1. All age groups reported more male than female cases except the age group 61 years with females accounting for 74(52%) of the 154 cases. Also, 7109 (90%) of the 7894 reported cases were aged 40 years and below (Table 1). We noted that 48 (1%) of the cases had missing ages or documented as unknown at the time of the review. The median age of reported yellow fever cases was 16 years (ranging from 0 to 92 years).

**Table 1:** Age and sex distribution of suspected yellow fever cases reported and cumulated age group proportions in Nigeria between September 2017 and 2 September 2019

Age Group	Male	Female	Total	Age proportion (%)	Cumulative proportion (%)	Female:male ratio
< 1 year	12 (57%)	9 (43%)	21	0	0	1.3:1
1–5 years	993 (62%)	617 (38%)	1610	20	20	1.6:1
6–10 years	821 (61%)	527 (39%)	1348	17	37	1.6:1
11–15 years	522 (61%)	338 (39%)	860	11	48	1.5:1
16–20 years	505 (54%)	429 (46%)	934	12	60	1.2:1
21–25 years	462 (53%)	410 (47%0	872	11	71	1.1:1
26–30 years	360 (52%)	332 (48%)	692	9	80	1.1:1
31–35 years	211 (52%)	198 (48%)	409	5	85	1.1:1
36–40 years	205 (56%)	158 (44%)	363	5	90	1.3:1
41–45 years	120(58%)	88 (42%)	208	3	93	1.4:1
46–50 years	100(53%)	90 (47%)	190	2	95	1.1:1
51–55 years	50 (55%)	41 (45%)	91	1	96	1.2:1
56–60 years	54 (57%)	40 (43%)	94	1	97	1.4:1
61 years +	80 (52%)	74 (48%)	154	2	99	1.1:1
Unknown/missing	33 (69%)	15 (31%)	48	1	100	2.2:1
Total	4528 (57%)	3366 (43%)	7894	100		1.3:1

*Descriptive statistics: Mean = 19.3 standard error = 0.17 median = 16 mode = 4 standard deviation = 15.4 range = 0–92 confidence level (95.0%) = 0.34

**Fig. 2:**
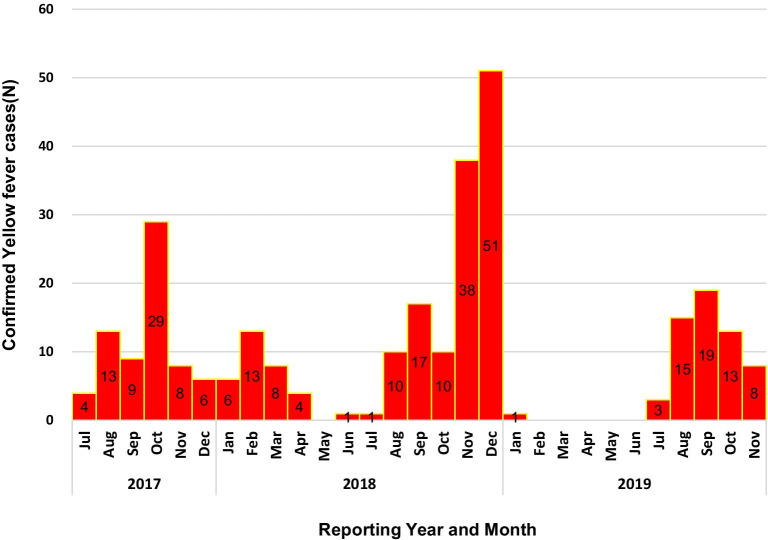
Epidemic curve showing laboratory-confirmed cases of yellow fever by month in Nigeria between September 2017–*2019

Figure 2 shows the epidemic curve of confirmed yellow fever cases between September of 2017 and 2019 shows a total of 287 laboratory confirmed cases with a monthly average of eight cases ranging from 0 to 51 cases. The highest number of cases was confirmed in November and December 2019 with 38 and 51 cases. Only 6 months during this period did not have a confirmed case (1 month in 2018 and 5 months in 2019). The curve shows peaks of confirmed cases in the last months of the year. The highest number of confirmed cases was reported in 2018 with 159 cases, while the least was reported in 2019 (January–November) with 59 cases.

**Fig. 3:**
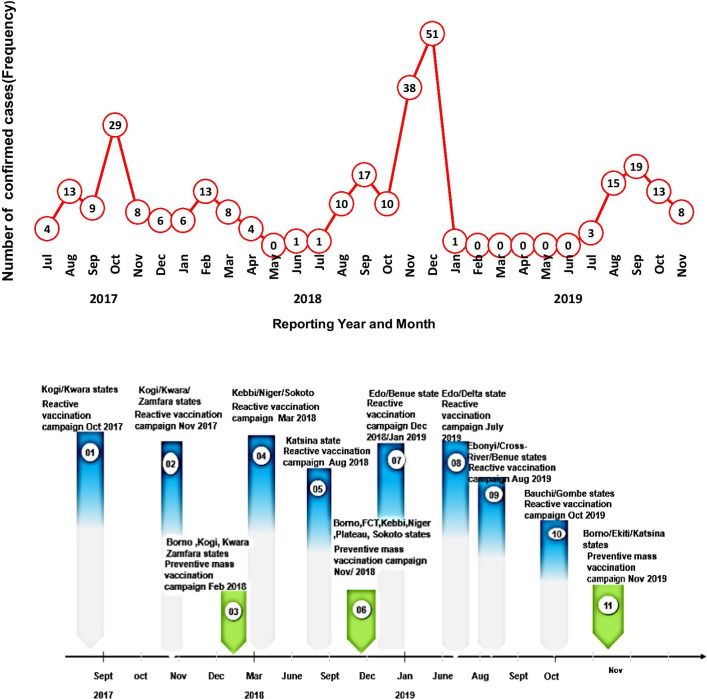
Map showing areas with yellow fever outbreaks in September 2017–2019 and epidemic blocks and spread in Nigeria. *Suspected and confirmed Yellow fever spatial distribution by state and LGA/District with clustering of outbreaks in identified epidemic blocks

Yellow fever cases were confirmed by laboratory testing through PCR or PRNT in 55 LGAs in 24 states of Nigeria from September 2017 to September 2019 (Fig. 3). The outbreaks of yellow fever can be divided into four epidemiologic blocks. Block 1 is the Kwara/Kogi block which spread northwards to Zamfara, Niger, Benue, Katsina, Kebbi and Kano. Block 2 is the Edo block which spread to affect Osun, Ekiti, Ondo and Delta states. Block 3 is the Ebonyi Block which spread to Abia, Anambra state and the fourth block is the Bauchi block which spread to Gombe, plateau and Borno states. These geographic areas served as distinct yellow fever epidemic epicentres, and no link of spread was reported between these different blocks.

**Fig. 4:**
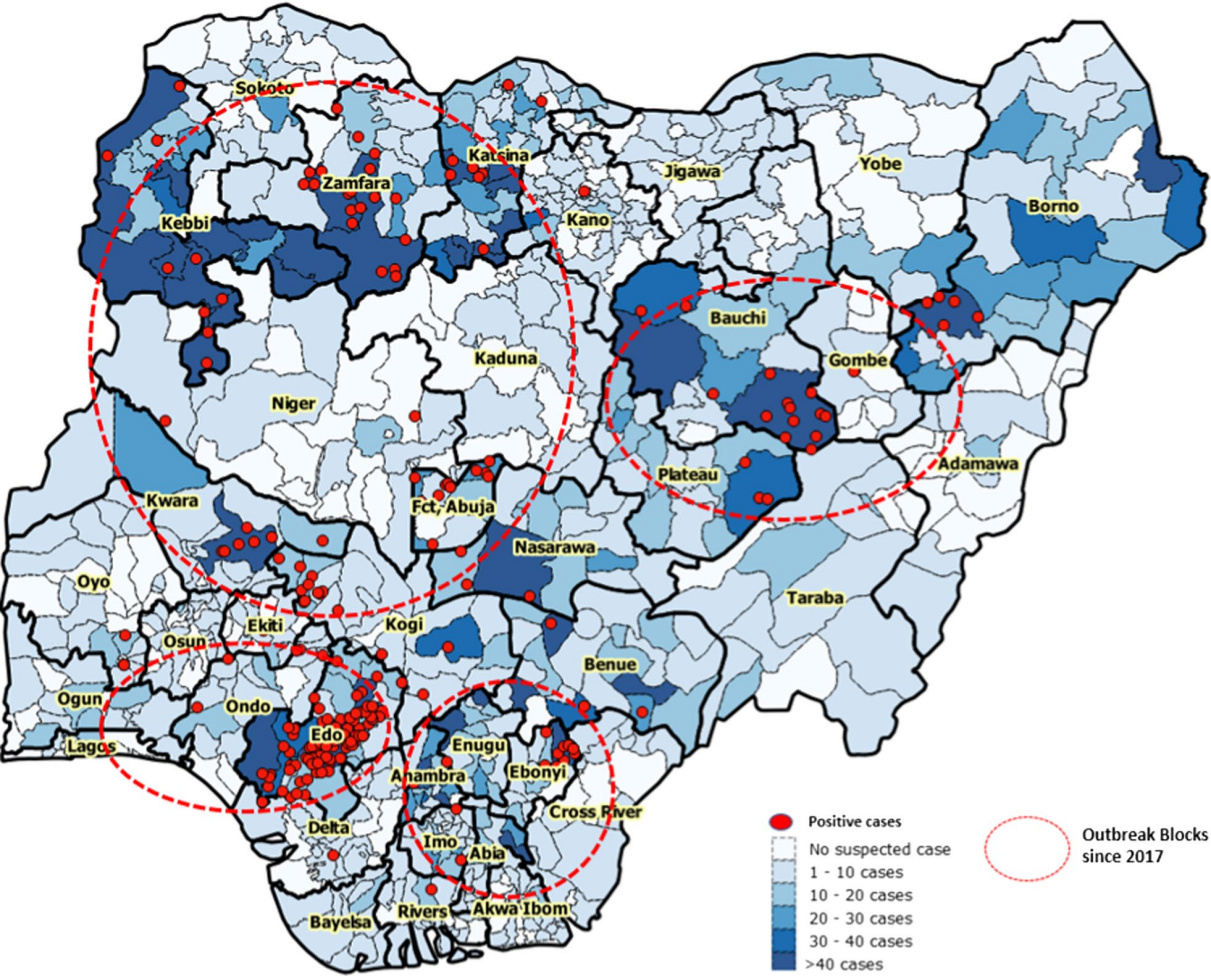
Bar chart showing average duration for various phases of the yellow fever detection and response by year and ICG application 2017–2019

The duration for responding to a yellow fever outbreak in Nigeria ranged from 15 to 132 days. The average duration between the confirmation of outbreaks to the implementation of the vaccination response in Nigeria between 2017 and 2019, as shown in Fig. 4 was 68 days. This average duration varied from 83 days in 2019, 43.5 days in 2018 and 42.3 days in 2017. The longest phase of this process (56%) is the period between confirmation and successfully submitting an ICG application. This phase took an average of 31 days. However, the average duration for ICG approval was 10 days and another 27 days) for vaccination implementation.

**Fig. 5:**
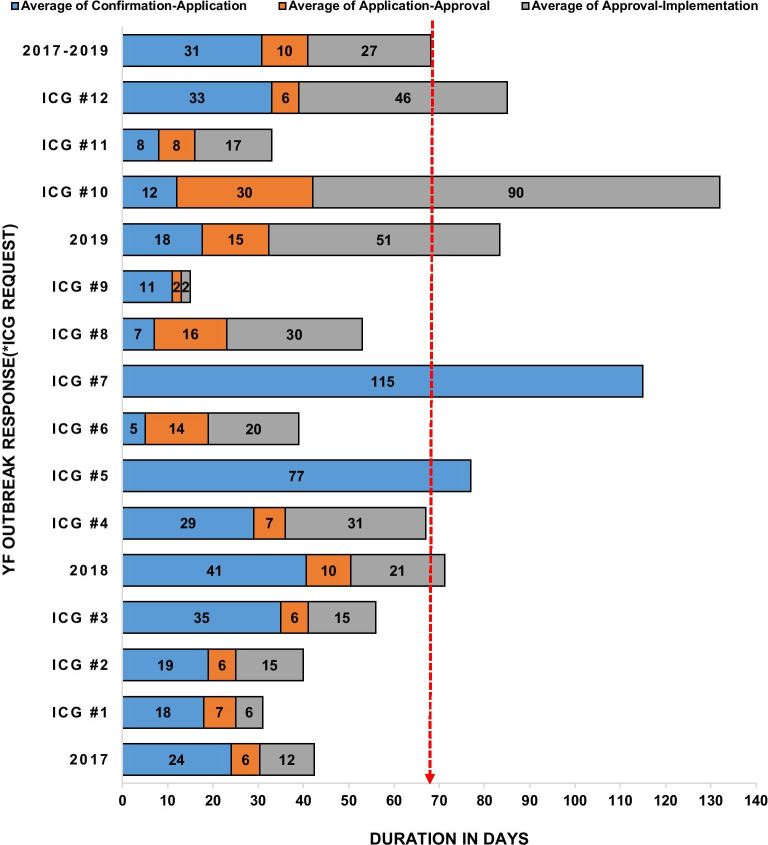
Case fatality rate of yellow fever by state and zone in Nigeria between 2017 and 2019

The case fatality rates during the yellow fever outbreaks reported between September of 2017–2019 are shown in Fig. 5. The highest CFR was reported from states in the south-south zone with reports of 11.7%. This high rate is accounted for mainly by Edo state with the highest CFR of 19.4% followed by the FCT, Abuja with a CFR of 13.9%. The national CFR for the yellow fever outbreak reported in Nigeria between September 2017–2019 is 2.6% amongst laboratory-confirmed cases.

**Fig. 6:**
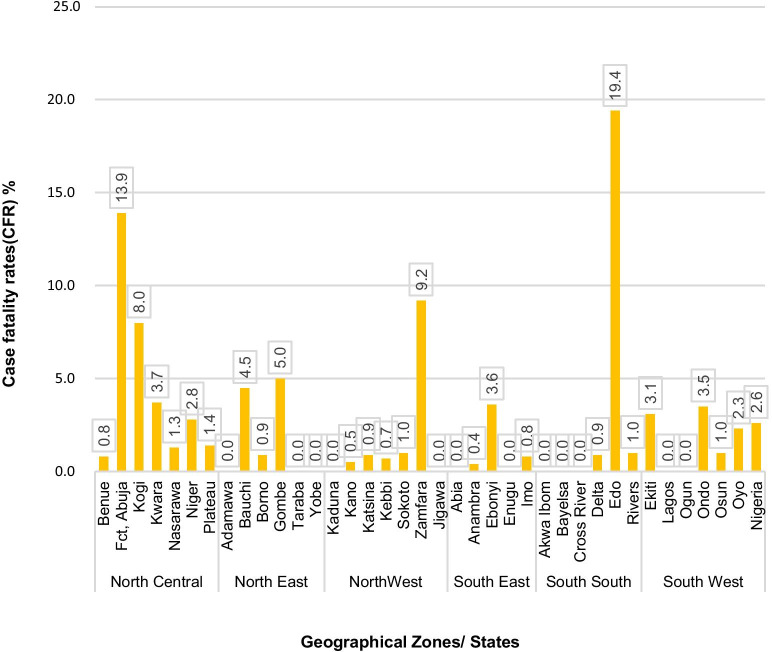
Trend line showing reported and confirmed yellow fever cases and vaccination campaigns in Nigeria between September 2017–2019

As shown in Fig. 6, 11 vaccination activities were conducted across multiple states in response to the multiple confirmed outbreaks and as part of the EYE strategy plan in Nigeria between the period under review. Nine targeted reactive vaccination campaigns were conducted in LGAs across 14 states of Katsina, Kebbi, Kogi, Kwara, Niger, Sokoto, Delta, Edo, Zamfara, Ondo, Benue, Cross River, Ebonyi and Bauchi. Also, three phased preventive mass vaccination campaigns were also implemented during this period covering FCT and Six states of Katsina, Kebbi, Niger, Sokoto, Borno and Plateau.

A total of 45,648,243 persons aged < 45 years were vaccinated in Nigeria through reactive and preventive mass campaigns between September 2017 and September 2019 (Table 2). Reactive vaccination accounted for 100% of all vaccinations in 2017. In 2018, 16% of all yellow fever vaccination campaigns conducted were through reactive campaigns while in 2019, this was 37%.

**Table 2:** Summary of vaccination interventions conducted in response to the yellow fever outbreak in Nigeria 2017–2019 through reactive and preventive campaigns in the four identified Epidemic blocks

Epid. Block	State	ICG requests and reactive vaccinations			Preventive mass vaccination campaigns (PMVC)					Total Vaccinated	
		Implementation months	Total reactive vaccinations	Post campaign coverage survey	Nov-18	Feb-18	Oct–Dec 2019	Total PMVC vaccinations	Post campaign coverage survey		
Block 1	FCT					3,269,041		3,269,041	96%	3,269,041	29,357,509
	Katsina	Aug-18	154,131	74%			6,720,113	6,720,113	84%	6,874,244	
	Kebbi	Mar-18	1,525,308	89%		2,283,993		2,283,993	62%	3,809,301	
	Kogi	Nov-17	776,995	92%	3,153,422			3,153,422	92%	3,930,417	
	Kwara	Nov-17	1,025,727	87%	1,884,065			1,884,065	85%	2,909,792	
	Niger	Mar-18	1,222,506	91%		4,342,518		4,342,518	78%	5,565,024	
	Sokoto	Mar-18	191,239	87%		2,808,451		2,808,451	69%	2,999,690	
Block 2	Delta	Jul-19	431,164	73%				0		431,164	7,814,903
	Edo	Jul-19	2,176,515	64%				0		2,176,515	
	Zamfara	Dec-17	1,250,404	80%	2,767,267			2,767,267	80%	4,017,671	
	Ondo	Jul-19	1,189,553	87%				0		1,189,553	
Block 3	Benue	Aug-19	710,308	78.%				0		710,308	1,854,461
	Cross River	Aug-19	129,713	NA				0		129,713	
	Ebonyi	Aug-19	1,014,440	NA				0		1,014,440	
Block 4	Bauchi**		407,708					0		407,708	6,621,370
	Borno*		0		983,982	524,378		1,508,360	78%	1,508,360	
	Plateau		0			4,705,302		4,705,302	94%	4,705,302	
	Total		12,205,711		8,788,736	17,933,683	6,720,113	33,442,532		45,648,243	

## Discussion

In our review of yellow fever outbreaks reported in Nigeria between September 2017 and September 2019, a total of 287 laboratory confirmed Yellow fever cases with a monthly average of eight cases ranging from 0 to 51 cases. We have documented a sustained outbreak across four major epidemic blocks around Kwara/Kogi, Edo, Ebonyi and Bauchi states with multiple cases. The peak of confirmed cases was recorded in November and December of 2018, which aligns with the most significant outbreaks reported in Edo State (Fig. 6). Smaller peaks were observed in October 2017 and August 2019. These peaks also aligned with the Kwara/Kogi outbreak in 2017 and the outbreak in Alkaleri LGA in Bauchi state in August 2019. Eight reactive yellow fever vaccination campaigns were conducted as a response to these outbreaks between this period and three phases of preventive mass vaccination campaigns vaccination since then with almost 45 Million persons vaccinated.

We noted a national case fatality rate during the 2-year period of 2.9% which was much lower than 53% previously reported rates in Brazil in 2017 but close to the range of 20–50% as stated by Gubler in unexposed populations [21]. We also found delays between outbreak confirmation and vaccination response. These delays in response to reported outbreaks have a big impact of the impact of outbreak response achieving the third strategic objective to contain outbreaks rapidly. While various interventions for control were implemented in Nigeria, there remained gaps in the timeliness and effectiveness of responses conducted between September 2017 and September 2019. While the implementation of the EYE strategy remains on track, we are concerned about achieving strategic objectives 2 and 3 (which focus on preventing the international spread and rapidly controlling outbreaks) if these gaps are not addressed.

The delays in the confirmation-response aspect documented show the need to improve efficiencies in the investigation and pursue a left shift in the epidemic curve to reduce further deaths and the associated health and economic costs which come with Yellow fever and other viral haemorrhagic outbreaks. The need for early reporting and rapid response has been documented by several scholars, as documented in the global health risk framework [22]. The lag times for the response to the outbreaks are similar to those documented before the institution of the revised IHR (2005) in 2007 by Chan et al. [23]. There is a need, therefore, for countries in the African region to further improve on disease reporting and outbreak discovery and shorting the time for response. Nigeria must improve on its ability to report any outbreaks through the multiple platforms available for disease reporting in Nigeria. The lessons from the polio programme can also be leveraged upon to ensure complete documentation and outbreak investigation as well as prompt laboratory testing and confirmation within the country. At the same time, other complementary tests can continue to be done internationally while not preventing the pulling the trigger for an intervention response. The most delays affecting timely response occur during the period between an outbreak confirmation and commencement of the reactive vaccination response. The delays in this time frame results from operational preparations and political endorsements for kick starting the campaigns. Other challenges include the administrative bottle necks for the distribution of finances and commodities to the subnational levels were the response is to be implemented. While some of these gaps can be addressed by improving reporting, investigation and confirmation, this does not address the prompt interventions required following approvals by the ICG for commencement a reactive vaccination campaign. Coordination of this activity needs to be improved upon between the two government agencies responsible (NPHCDA and NCDC) as well as prepositioning of resources for a qualitative response at national and state levels. This will include review and updates of the yellow fever outbreak protocols and the availability and functionality of technically competent rapid response teams and the emergency stockpiles. These efforts could play a huge role in reducing response time to outbreaks to < 2 weeks. All these efforts focus on the third objective of the EYE strategy.

We also noted that the current outbreaks have shown anthropogenic sylvatic cycles and village epidemics around villages and forested vegetation. This is shown in the four epidemic blocks in Bauchi, Ebonyi, Edo and Kwara/Kogi unlike the epidemics reported in Brazil, we, however, could not demonstrate a natural sylvatic transmission and the role of non-human primates in maintain yellow fever transmission [24]. The recent outbreak reported in September 2019 in Alkaleri LGA of Bauchi state which hosts the Yankari game reserve further reiterates the need to assess the role of non-human primates in the outbreaks in Nigeria and the need for detailed studies typing non-human primates as well as specific vectors involved in the yellow fever epidemiology in Nigeria.

No documentation in this study has been made on the efforts relating to the second objective of the EYE strategy relating to prevention of international spread in Nigeria. Reducing the risk of international spread relate to the efforts around ports of entries. Nigeria must continue to improve efforts at ensuring all persons at borders and airports are vaccinated with the yellow fever vaccine and have the right documentation to this effect. Progress in documenting this has recently been seen with the electronic records as recently launched in Nigeria. The e-yellow fever card allows for validation of persons vaccinated as well as address the issues around fake cards reported in the country. Nigeria must also continue to educate the populace to understand the importance of being vaccinated and not the possession of the yellow fever card as for prevention of the disease.

Our paper however has a few limitations. Firstly, the analysis is based on data available as part of the yellow fever surveillance system in Nigeria at the national level. We acknowledge the heterogenicity of reporting systems quality across multiple states and the available competencies and capacities for response in the different states. This is even so despite one national guideline by the NCDC for outbreak investigation and control. We are aware that efforts at national level to strengthen these gaps cut across all states and may not influence the outcomes and conclusions in this paper. Secondly, we have not factored in the various activities and outbreaks that have occurred in Nigeria within this same time frame which may have affected some of the time frames documented in this paper. All documented dates of activity implementation are based on available reports submitted by the states and communication at national levels. We are aware that the surveillance and immunization system and personnel at lower levels are the same for all diseases and multiple VPD outbreaks may affect timeliness of reporting and response. We have not set out to document any reasons for delays or comparison within states. Further assessment of challenges or barriers to timely response to outbreaks may help in improving timeliness of outbreak reporting, investigation and response.

## Conclusion

We documented the yellow fever outbreaks experienced in Nigeria between September 2017 and 2019 and reviewed the epidemiological information and assessment of the response including vaccination during this period.

Nigeria experienced intermediate outbreaks of yellow fever which occurred in four epidemic blocks with 7894 suspected cases and 287 laboratory confirmed cases. While over 45 million persons were vaccinated with the yellow fever vaccine during this period, the average time of commencement of reactive vaccination response following outbreak confirmation was 68 days.

Gaps remain in the quality and timeliness of response. More efforts towards improving reporting and response times as well as preparedness efforts are needed to ensure a left shift in the epidemic curve and prevent morbidity and mortality from the yellow fever disease. A reduction in the documented average response time of 68 days can play a critical role in achieving this left shift.

Efforts towards improving population immunity through vaccinations (routine and mass campaigns) remain critical in preventing yellow fever outbreaks. These interventions, alongside others recommended as part of the EYE strategy, would go a long way in ensuring that Nigeria and other countries in Africa achieve the strategic target of eliminating yellow fever epidemics by 2026.

## Data Availability

All data sets analysed and included in this paper are based on programme data and reports. The datasets used and/or analysed for this paper are available from the corresponding author on reasonable request.

## Abbreviations

EYE: Eliminating Yellow Fever epidemics
FCT: Federal Capital Territory
GoN: Government of Nigeria
ICG: International Coordinating Group
IDSR: Integrated disease surveillance and response
IMS: Incident management system
IP Dakar: Institute Pasteur Dakar
LGA: Local Government Area
NCDC: Nigeria Center for Disease control
NPHCDA: National Primary Health Care Development Agency
NHP: Non-human primates
PCR-rt: Polymerase chain reaction-real time
PRNT: Plaque reduction neutralization test
PMVC: Preventive Mass Vaccination Campaign
PHEOCs: Public Health Emergency Operation Centers
RRL: Regional Reference Laboratory
UNICEF: United Nations Children Education Fund
WHO: World Health Organization
WUENIC: WHO and UNICEF estimates for national immunization coverage
